# Self-reported rather than registered cancer is associated with psychosocial strain

**DOI:** 10.1186/1471-2296-13-107

**Published:** 2012-11-13

**Authors:** Sonja Korpimäki, Markku Sumanen, Sakari Suominen, Kari Mattila

**Affiliations:** 1 Tampere Health Centre, Tampere, Finland; 2 Medical School, University of Tampere, Tampere, Finland; 3 Department of Public Health, Nordic School of Public Health, University of Turku, Turku, Finland; 4 Centre of General Practice, Hospital District of Pirkanmaa, Tampere, Finland

**Keywords:** Cancer, Psychosocial factors, Self-reported disease

## Abstract

**Background:**

Individuals with only a subjective experience of cancer may conceal severe psychological distress and act like patients with verified disease. The purpose of the study was to establish whether some typical psychosocial factors may be linked to subjects with registered cancer (*confirmed*) and also to those with self-reported cancer lacking accompanying registered data (*non-confirmed*).

**Methods:**

The material comprised 25 898 working-aged individuals (response rate 40.0%) in 1998. Of these 19 629 also responded at the follow-up in 2003 (response rate 75.8%). The analyses focused on respondents with cancer diagnosis in 2002 or earlier according to data of the Finnish Cancer Registry and self-report of cancer in 2003 (*confirmed*) (N=330) and on respondents with self-reported cancer only but lacking registered diagnosis (*non-confirmed*) (N=140). Those who neither reported cancer nor had a diagnosis were included as a control group (N= 18 299).

**Results:**

Respondents with *confirmed* cancer belonged more often to the oldest age-group than those with *non-confirmed* cancer. Respondents with *non-confirmed* cancer were more often obese, depressed and reported less social support compared to subjects with *confirmed* cancer. Compared to controls they had a statistically significantly increased risk of depression, lower optimism, lower life satisfaction, more childhood adversities, more negative life events and less social support.

**Conclusions:**

Individuals with only a subjective experience of cancer reported more psychosocial strain than those with accompanying registered cancer. Self-report of a severe disease like cancer without corresponding clinical findings might reflect heavy psychological distress which should be taken into consideration in clinical work.

## Background

There are about 200 000 people in Finland, about 4% of the population, who have or have had cancer [1]. The yearly incidence of cancer in 2008 was for males 283.6 per 100 000 person-years and for females 249.9 per 100 000 person-years. The most common cancer in the industrialized countries is prostate cancer in males (4 200 new cancer cases in Finland in 2008) and breast cancer in females (4 300 new cancer cases in Finland in 2008).

The development of cancer is a long-term gradual process taking years or even decades. Cancer is a multiform disease state with very different clinical manifestation. An increasing proportion of cancer patients recover. The common conception, nonetheless, is that cancer is a very serious possibly life threatening condition turning it to a subjectively stressful experience.

Thus, individuals with only a subjective experience of cancer may conceal severe psychological distress, potentially based on misunderstanding of the judgements given by the doctor or another representative of the health care system. These individuals may act as patients with verified disease. The purpose of the study was to establish whether some typical psychosocial factors may be linked to subjects with registered (*confirmed*) cancer and also to those with self-reported cancer lacking accompanying register data (*non-confirmed*).

## Methods

The Health and Social Support Study (HeSSup) is a prospective follow-up study focussing on psychosocial determinants of health of the Finnish working-age population. The subjects belonged to a random sample drawn from the Finnish Population Register [2], stratified into four age-groups: 20–24, 30–34, 40–44 and 50–54 at baseline. In 1998 a completed postal questionnaire was returned by 25 898 participants (response rate 40.0%). Of these 19 629 also responded at the follow-up in 2003 (response rate 75.8%). Analysis of non-responders showed a good compatibility of the HeSSup sample with the Finnish population [3,4]. The concurrent joint Ethics Committee of the University of Turku and the Turku University Central Hospital considered approval not necessary for a normal cohort study, but all participants were requested to sign a consent form containing information about the study and to grant permission to allow subsequent studies with the same data set and possibility to link with national health registries.

The Finnish Cancer Registry maintains a nationwide database on all cancer cases in Finland from 1953 onwards [5]. It is also an internationally active institute for statistical and epidemiological cancer research. There were 1 037 subjects in the HeSSup sample with a cancer diagnosis between the years 1955 and 2006 when the survey data was linked with data of the Cancer Registry. Of them 263 (basalioma, polycythemia vera, myelofibrosis etc.) were excluded as in the basic statistics of the Cancer Registry [6]. Thus 774 subjects having malignant cancers were included in the study. To be able to use the information within the HeSSup sample, only participants having a tumour diagnosed in 2002 or earlier were included in the analysis (N=396). This limitation was imposed so that recently diagnosed cancer would not influence the data collected with the 2003 questionnaire.

**Figure 1:**
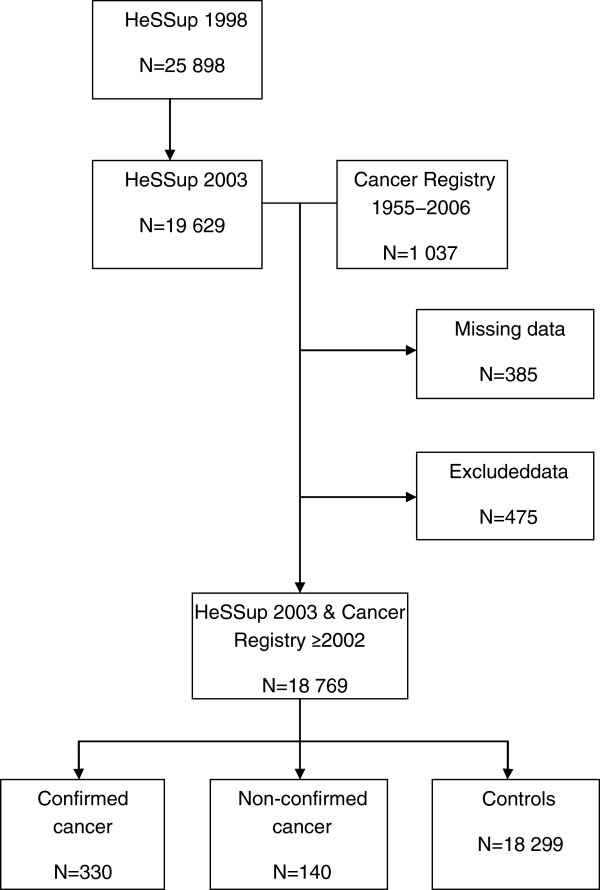
Flowchart representing the study population and the formation of the subjects.

In the HeSSup study the participants were asked whether or not a doctor had told them that they have or have had a malignant tumour (cancer). The analyses focused on respondents who had registered cancer and also self-reported it in the follow-up survey in 2003 (N=330), termed “*confirmed cancer*”, and on those who self-reported cancer only but lacked an accompanying registry diagnosis ever (N=140), termed “*non-confirmed cancer*” here (Figure 1). Those who neither reported a cancer nor had a diagnosis were included as controls (N= 18 299).

This study has a cross-sectional design. The analyses compared demographic, life-style and psychosocial factors asked in 2003 between respondents having *confirmed* cancer and *non-confirmed* cancer, and controls. Respondents were categorized according to gender and four age-groups: 25–29, 35–39, 45–49 and 55–59 in 2003. Education, as a proxy for socioeconomic status, was ascertained by a self-report on the matriculation examination (≥12 year education). Marital status was classified into two groups: married or cohabiting and single, widow or divorced. Living was graded as alone or together with one or more persons. Obesity was measured by the body mass index (BMI <30 or ≥30 kg/m^2^). Participants were asked whether or not they had ever smoked and current smoking was categorized as <5 or ≥5 cigarettes per day. Alcohol consumption was calculated as grams of alcohol per week. Weekly consumption of pure alcohol under 22 g was categorized as none or minimal, more than 22g but less than 175g in women and under 263g in men as moderate and more as heavy. Exercise activity was categorized as little (MET <2) or much (MET ≥2) according to daily exercise [7].

The general sense of stressfulness was measured by the Reeder Stress Inventory [8]. This comprises the following four statements: “In general I am usually tense or nervous”, “There is a great amount of nervous strain connected with my daily activities”, “At the end of the day I am completely exhausted mentally and physically” and “My daily activities are extremely trying and stressful.” Participants indicate the extent to which each statement applies to them using a 5-point Likert scale. The score was calculated as the overall sum of the four items (range 4–20). The sum was classified into three: little (≥17), moderate (12–17) or great (≤12).

Symptoms of depression were assessed by the widely used 21-item Beck Depression Inventory [9]. A sum score over the cut-off value of 19 was taken to indicate (moderate or severe) depressiveness.

Hostility was assessed by a three-item expressed hostility scale [10]. This included respondents’ self-ratings of irritability, ease of anger-arousal, and argumentativeness, which were rated on a 5-point Likert scale. The hostility scale was built up by the sum score on these three items (total range 3–15) and categorized into three classes (0–5, 6–12 or 13–15).

The level of optimism was measured by the Life Orientation Test-Revised (LOT-R) [11]. This is a 6-item scale covering generalized future expectations; three items are worded positively and three negatively, and the items are presented as a 5-point Likert scale. The LOT-R scale was reconstructed by summing (total 6–30) and categorized into three classes (<20, 20–24 or >24).

Life satisfaction was determined by the responses to four questions dealing with interest in life, happiness, general ease of living and loneliness [12]. The range of the sum score for life satisfaction (LS) was 4–20. An increase in values indicated a decrease in life satisfaction: satisfied (LS 4–6), intermediate group (LS 7–11) or dissatisfied (LS 12–20).

Subjects were asked to recall their childhood adversities in terms of six questions: “Did your parents divorce?”, “Did your family have long-lasting economic difficulties?”, “Did serious conflicts arise in your family?”, “Were you often afraid of some member of your family?”, “Was someone in the family seriously or chronically ill?” and “Did someone in the family have problems with alcohol?” [13]. Alternative answers were Yes, No or I do not know. Only the first two options were included in the statistical analyses. The number of childhood adversities per individual was calculated (0, 1 or 2–6).

Respondents were also requested to recall life events. Adverse events were asked via a 21-item question sequence considering e.g. death or illness of close person, divorce, loss of job, accidents and violence [14,15]. The sum was classified into two groups: few (0–2) or many (>2). Likewise positive events were ascertained from answers to the question “Has some remarkable positive experience or event happened to you?” The eight response options were: in family life, in working life, in human relationships, in spiritual life, in economic situation, in living, in hobbies and in love. The sum was classified into two groups: few (0–2) or many (>2).

Social support was estimated by the Brief Social Support Questionnaire [16]. It comprises six questions: “Whom can you really trust when you want to forget your sorrow when feeling stressed?”, “Whose help can you really count on when needing relaxation under pressure?”, “Who really accepts you with all your strengths and weaknesses?”, Who really cares about you whatever happens?”, “Who can you really trust to get you to feel better when upset?” and “Whom can you count on to comfort you when you are screwed up?”. Each of the questions gave zero to six sources of support to select from. The sum score (0–36) was classified: 0–5, 6–11, 12–17 or ≥18. Low social support was indicated by the lowest class. The type of social support was based on the mean of options: “I get more support than I give”, “I give more support than I get” or “I get as much support as I give” to questions considering the three most important adults in question.

The statistical significance of differences between respondents having *confirmed* and *non-confirmed* cancer was tested by χ2 test. Odds ratios (OR) with 95% confidence interval (CI) for cancer in the multivariate logistic regression analysis were calculated in both groups for demographic, life-style and psychosocial factors with adjustment for age and sex. The analyses were made using SPSS for Windows, release 13.

## Results

The majority of the study population were women, while age was evenly distributed (Table 1). A minority among the respondents had a higher educational level. Most were married or cohabiting and lived with one or more persons. Their life-style was on average healthy: BMI equal to 30 or over marking obesity was noted in one respondent out of four, regular smoking was reported by one in five, heavy alcohol drinking by one in twenty and little exercise by one in four. Moderate or severe depression came up in 4.3% of reports. The feelings of stress, hostility, optimism and satisfaction with life were mostly expressed as moderate. Over half of the respondents graded their social support in the first or second category, indicating minor support. The prevailing type of social support was “getting more than giving”. No childhood adversities had been experienced by 39%; on the other hand nearly the same proportion had experienced many. Of all respondents 78% reported only few negative events. Many positive events were reported by 88%.

**Table 1:** Distribution of demographic, life-style and psychosocial factors among the study population (N=18 769)

	**N**	**%**
*Gender*		
Female	11 519	61.4
Male	7 247	38.6
*Age-group*		
55-59	4 984	26.6
45-49	4 649	24.8
35-39	4 336	23.1
25-29	4 797	25.6
*Education*		
Lower	10 412	55.5
Higher	8 332	44.5
*Marital status*		
Marriage/cohabitation	12 838	68.5
Single/widow/divorced	5 909	31.5
*Living*		
With ≥1 persons	15 116	81.5
With 0 persons	3.439	18.5
*Obesity*		
BMI < 30	13 917	75.5
BMI ≥ 30	4 519	24.5
*Smoking*		
No	8 227	47.8
Quitted	5 163	30.0
Regular (<5 cigarettes/day)	416	2.4
Regular (≥5 cigarettes/day)	3 410	19.8
*Alcohol use*		
None or minimal	6 161	32.9
Moderate	11 523	61.5
Heavy	1 043	5.6
*Exercise activity*		
Much (met≥2)	13 430	72.0
Little (met<20	5 222	28.0
*Stress*		
Low	5 113	27.5
Moderate	9 242	49.8
High	4 217	22.7
*Depression*		
Beck 0-19	17 631	95.7
Beck ≥20	787	4.3
*Hostility*		
Low	3 913	21.0
Moderate	10 483	56.2
High	4 245	22.8
*Optimism*		
High	5 747	30.9
Moderate	9 242	49.7
Low	3 590	19.3
*Life satisfaction*		
High	4 568	24.5
Moderate	10 553	56.7
Low	3 498	18.8
*Childhood adversities (n)*		
0	7 325	39.2
1	4 863	26.0
2-6	6 513	34.8
*Negative events*		
Few (0-2)	14 515	77.7
Many (>2)	4 158	22.3
*Positive events*		
Many (>2)	16 216	87.9
Few 90-2)	2 232	12.1
*Social support (points)*		
≥18	1 682	9.0
12-17	5 008	26.7
6-11	10 212	54.4
0-5	1 867	9.9
*Support type*		
Gets as much as gives	6 010	32.5
Gets more than gives	7 170	38.8
Gets less than gives	5 284	28.6

There were no statistically significant differences in gender between respondents having *confirmed* and *non-confirmed* cancer but those with *confirmed* cancer belonged more often to the oldest age-group analyzed (p<0.001) (Table 2). The sociodemographic indicators education, marital status and number of people living in the same household showed no differences. The subjects with *non-confirmed* cancer were more often obese (p=0.003) than those with *confirmed* cancer. Life-style factors such as smoking, alcohol consumption and exercise activity were equally reported. Depression was more common among subjects having *non-confirmed* cancer than among those with *confirmed* cancer (p=0.044). This group also reported less social support (p<0.001) but its type showed no difference. Likewise reporting of other examined psychosocial factors, including number of life events, did not differ.

**Table 2:** Distribution of demographic, life-style and psychosocial factors among respondents having registered (confirmed cancer) and only self-reported cancer (non-confirmed cancer)

	**Confirmed**	**Non-confirmed**	**P-value**
	**N=330 %**	**N=140 %**	
*Gender*			0.687
Female	73.9	72.1	
Male	26.1	27.9	
*Age-group*			<0.001
55-59	58.8	42.9	
45-49	27.3	26.4	
35-39	8.8	20.7	
25-29	4.8	10.0	
*Education*			0.296
Lower	64.5	69.6	
Higher	35.5	30.4	
*Marital status*			0.806
Marriage/cohabitation	70.3	71.4	
Single/widow/divorced	29.7	28.6	
*Living*			0.947
With ≥1 persons	76.5	76.8	
With 0 persons	23.5	23.2	
*Obesity*			0.003
BMI < 30	70.9	56.7	
BMI ≥ 30	29.1	43.3	
*Smoking*			0.105
No	46.8	42.4	
Quitted	35.1	28.8	
Regular (<5 cigarettes/day)	1.6	2.4	
Regular (≥5 cigarettes/day)	16.6	26.4	
*Alcohol use*			0.408
None or minimal	40.1	40.7	
Moderate	55.7	52.1	
Heavy	4.3	7.1	
*Exercise activity*			0.593
Much (met≥2)	71.0	68.6	
Little (met<2)	29.0	31.4	
*Stress*			0.102
Low	27.7	24.6	
Moderate	51.1	44.8	
High	21.2	30.6	
*Depression*			0.044
Beck 0-19	93.8	88.2	
Beck ≥20	6.2	11.8	
*Hostility*			0.415
Low	24.2	19.3	
Moderate	56.0	62.1	
High	19.9	18.6	
*Optimism*			0.096
High	32.8	23.9	
Moderate	46.0	47.8	
Low	21.2	28.4	
*Life satisfaction*			0.466
High	18.7	19.6	
Moderate	58.6	52.9	
Low	22.7	27.5	
*Childhood adversities (n)*			0.293
0	33.7	26.4	
1	27.7	30.0	
2-6	38.6	43.6	
*Negative events*			0.515
Few (0-2)	72.3	69.3	
Many (>2)	27.7	30.7	
*Positive events*			0.456
Many (>2)	83.8	86.6	
Few (0-2)	16.2	13.4	
*Social support (points)*			<0.001
≥18	8.2	5.7	
12-17	29.7	12.9	
6-11	53.6	63.6	
0-5	8.5	17.9	
*Support type*			0.231
Gets as much as gives	41.1	35.5	
Gets more than gives	27.9	25.4	
Gets less than gives	31.0	39.1	

In the multivariate regression analyses with adjustment for age and sex, single, widowed or divorced respondents and also respondents living alone had more *confirmed* cancer than controls (Table 3). Subjects whose educational level was lower had increased likelihood to have *non-confirmed* cancer. Single, widowed or divorced respondents also had more *non-conformed* cancer whereas those who lived alone had not. Obesity seemed to increase the risk for reporting *non-confirmed* cancer twofold. Regular and heavier smoking likewise increased this risk. Neither alcohol use nor exercise activity had effects on the likelihood of *confirmed* or *non-confirmed* cancer.

**Table 3:** ORs (95% CI) of demographic and life-style factors reported among respondents in the multivariate logistic regression analysis for registry-confirmed (confirmed cancer) and only self-reported cancer (non-confirmed cancer) with adjustment for age and sex

	**Confirmed ~ control**		**Non-confirmed ~ control**	
	**OR**	**95 % CI**	**OR**	**95% CI**
*Education*				
Higher	1		1	
Lower	0.913	0.719 - 1.159	1.523	1.039 - 2.232
*Marital status*				
Marriage /cohabitation	1		1	
Single/widow/divorced	1.384	1.083 - 1.768	1.596	1.119 - 2.274
*Living*				
With ≥1 persons	1		1	
With 0 persons	1.366	1.050 - 1.777	1.431	0.957 - 2.138
*Obesity*				
BMI < 30	1		1	
BMI ≥ 30	1.024	0.799 - 1.311	2.120	1.497 - 3.003
*Smoking*				
No	1		1	
Quitted	1.219	0.941 - 1.579	1.105	0.718 - 1.700
Regular (<5 cigarettes/day)	0.910	0.369 - 2.249	1.326	0.411 - 4.277
Regular (≥5 cigarettes/day)	0.989	0.713 - 1.372	1.652	1.062 - 2.571
*Alcohol use*				
None 0r minimal	1		1	
Moderate	0.835	0.661 - 1.055	0.754	0.527 - 1.077
Heavy	0.655	0.373 - 1.151	1.100	0.553 - 2.190
*Exercise activity*				
Much (met≥2)	1		1	
Little (met<2)	0.904	0.708 - 1.153	1.083	0.755 - 1.553

*Confirmed* cancer was seen only in the context of low life satisfaction and many negative life events in the background (Table 4). In the case of *non-confirmed* cancer, however, a statistically significantly increased risk was seen in the context of most psychosocial factors analyzed. The likelihood in cases of depression was 2.75 (95% CI 1.62−4.66), in cases with low optimism 2.02 (95% CI 1.26−3.24) and with low life satisfaction 1.80 (95% CI 1.20−2.96) compared to controls. Reported childhood adversities were associated with *non-confirmed* cancer; both in the case of one or 2–6 adversities. Adverse life events showed an association with report of *non-confirmed* cancer similar to that with *confirmed* cancer. Little social support was associated with reporting *non-confirmed* cancer (OR 2.69, 95% CI 1.20−6.06), but the interrelationship between receiving and giving support showed no association.

**Table 4:** ORs (95% CI) of psychosocial factors reported among respondents in the multivariate logistic regression analysis for registry-confirmed (confirmed cancer) and only self-reported cancer (non-confirmed cancer) with adjustment for age and sex

	**Confirmed ~ control**		**Non-confirmed ~ control**	
	**OR**	**95% CI**	**OR**	**95% CI**
*Stress*				
Low	1		1	
Moderate	1.090	0.840 - 1.415	1.052	0.686 - 1.612
High	0.964	0.701 - 1.326	1.553	0.979 - 2.463
*Depression*				
Beck 0-19	1		1	
Beck ≥20	1.291	0.813 - 2.051	2.749	1.620 - 4.664
*Hostility*				
Low	1		1	
Moderate	0.998	0.763-1.306	1.279	0.716 - 1.724
High	1.144	0.816-1.605	1.068	0.616-1.850
*Optimism*				
High	1		1	
Moderate	0.877	0.682 - 1.129	1.256	0.820 - 1.924
Low	1.126	0.827 - 1.533	2.018	1.258 - 3.239
*Life satisfaction*				
High	1		1	
Moderate	1.236	0.923 - 1.657	1.117	0.716 - 1.724
Low	1.510	1.069 - 2.131	1.800	1.096 - 2.957
*Child adversities (n)*				
0	1		1	
1	1.085	0.818 - 1.439	1.594	1.022 - 2.486
2-6	1.211	0.935 - 1.570	1.761	1.167 - 2.655
*Negative events*				
Few (0-2)	1		1	
Many (>2)	1.486	1.160 - 1.903	1.616	1.124 - 2.322
*Positive events*				
Many (>2)	1		1	
Few (0-2)	1.087	0.801 - 1.475	0.994	0.600 - 1.648
*Social support (points)*				
≥18	1		1	
12-17	1.195	0.775 - 1.845	0.770	0.334 - 1.775
6-11	0.839	0.553 - 1.272	1.748	0.841 - 3.635
0-5	0.689	0.999 - 1.181	2.693	1.197 - 6.060
*Support type*				
Gets as much as gives	1		1	
Gets more than gives	1.254	0.953 - 1.651	1.071	0.687 - 1.669
Gets less than gives	0.804	0.618 - 1.045	1.315	0.889 - 1.946

## Discussion

According to our findings psychosocial strain appeared to accumulate among subjects who had only subjective, i.e. *non-confirmed* cancer. They had an increased risk of depression, lower optimism, lower life satisfaction, more childhood adversities, more negative life events and less social support as compared to individuals with by means of registry data confirmed cancer. Thus, the results gave support for the pre-assumption that individuals with a subjective experience of cancer that has not been a provisional diagnosis nor has been based on missing or invalid registry data may conceal severe psychological distress. That kind of situation might partially be based on misunderstanding of the message given by a health care professional. They also had more often a lower educational level, were single, widowed or divorced, and were obese and regular heavier smokers.

### Strengths

The HeSSup sample may be regarded as sufficiently large and the response rate to the follow-up as high (75.8%). According to the kappa coefficient the consistency of responses on childhood adversities between the years 1998 and 2003 was good [17,18]. The consistency of some other psychosocial variables asked may be comparably stable, whereas some other self-reports may be more prone to reactivity. The measured variables can be considered valid, since they are widely used in well organized and referred studies [8-16].

The cancer diagnoses in our material can be considered reliable and extensive. The data quality and quality control of the Finnish Cancer Registry is valid [19], and it is highly confident that all diagnosed cancer cases were registered and those who only self-reported cancer did not have a verified diagnosis. The agreement between questionnaire data and medical records has proved to be good in chronic diseases with clear diagnostic criteria, while for diseases with no established diagnostic criteria it seemed poor [20]. When only definite diagnoses were included, questionnaire responses indicated a higher prevalence.

### Limitations

Misinterpretations of cancer diagnoses may have originated from doctors’ wording when questioning or excluding something during the examination, e.g. nevus extraction, prostate sample or Pap test. A provisional diagnosis is not subsequently confirmed upon more extensive work-up, after the patient was told that cancer was present. To reduce false interpretations, cancer types like basalioma, polycythemia vera and myelofibrosis were excluded, as they are in the basic statistics of the Cancer Registry. These cancers are usually only monitored, not actively treated. The word cancer may even today sound frightening. Hunziker and associates concluded that one in three “asymptomatic” patients had one or more hidden reasons for requesting a check-up [21]. One concern for a consultation might be a fear of cancer.

The analyses were adjusted for age and sex, but one further notable variable for adjustment might have been depression. The diagnosis of cancer was associated with depression, but the extent has varied in previous studies [22-24]. In our study depression was associated with reporting *non-confirmed* cancer, not with *confirmed* disease. Romanov and associates found that somatic disease, stressful life events and lack of social support each independently increased the risk of depressiveness [15]. Strömberg and associates found that perceived physical health, stress, family relations and work situation are relevant indicators in detection of patients at risk of depression in primary care settings [25]. One limitation in our study was the cross-sectional assessment of these and other psychosocial variables in the questionnaire.

To avoid the impact of recently diagnosed cancer on responses to the questionnaire in 2003, only cancers diagnosed in 2002 or earlier were included. No causal relationship between the psychosocial variables and the cancer diagnosis can be assumed. Here we could not know when e.g. negative life events have occurred, or whether the diagnosis changed life satisfaction or the feeling of social support. Factors such as time since diagnosis and treatment, type of treatment or possible cancer recurrence were not included in the analyses. This notwithstanding, Parker and colleagues found that disease characteristics appeared to have less impact on patients’ quality of life than did demographic variables or social support [23]. Hamer and associates showed that the presence of participants with a cancer history in community-based cohorts may lead to overestimation of the association between psychosocial distress and subsequent cancer mortality [26].

### Comparison to other studies

Psychosocial factors may contribute to cancer via high-risk life style factors. No such notable effect was seen either in our study or in the work of Chida and associates [27]. An increased likelihood of *a confirmed* cancer diagnosis was seen only in the context of low life satisfaction and a great number of negative life events in the background.

Socioeconomic status was measured in terms of education, which was lower among respondents who had *non-confirmed* cancer. Pukkala and Weiderpass found cancers of the cervix uteri and vagina to be associated with lower social class, whereas breast cancers were most common in higher social classes [28]. Living without a close relationship increased the risk of *confirmed* cancer here. Partner support was associated with high optimism and less despair in cancer patients [29]. Mortality has been estimated to be lowest at about 22–25 kg/m^2^ BMI and each 5 kg/m^2^ higher BMI has been associated with 10% higher mortality due to malignancies [30]. In our study obesity was associated only with reporting *non-confirmed* cancer. Smoking as a known risk factor for many types of cancer also replicated this finding, but not for *confirmed* disease. This was not surprising, since after confronting a disease people might be more prone to change their habits. In a recently published survey covering life-style behaviours among cancer survivors, males did not differ from controls and females were only more physically inactive [31].

Traeger and associates found that prostate cancer patients who experienced more daily stress may have poorer resources to cope with ongoing disease concerns [32]. In our study no difference in reported stress was seen between both groups of cancer patients and controls. The better psychosocial adaptation in women with breast cancer under treatment may affect biological parameters and may contribute to their health status [33]. Baider and associates found that cancer patients and also partners experiencing high psychosocial distress reported lower levels of perceived family support [34]. Our results are in accordance with this. Quality of life among cancer patients was shown to correlate with psychosocial variables, whereas no clear causation was seen in the work of Shapiro and associates [35]. Lebel and associates concluded that earlier stress-related problems during diagnosis and treatment predicted long-term stress-related problems among breast cancer patients [36].

Somatoform disorders like hypochondria may also explain the subjective experience of cancer. Haftgoli and associates examined the association with psychosocial stressors and depression, anxiety and somatoform disorders in primary care patients consulting GP for a physical complaint [37]. Stressors appeared to have less impact on levels of somatoform disorders than on depression or anxiety.

## Conclusions

Individuals with only a subjective experience of cancer reported more psychosocial strain than those with accompanying registered cancer. People with experience of having cancer but no verified diagnosis may act like cancer patients. Their need for health care services might increase. Doctors should not undertake groundless examinations or make even hazardous, harmful or injurious decisions. Instead such patients should be given support and their possible depressiveness should be treated. Self-report of a severe disease like cancer without corresponding clinical findings might reflect heavy psychological distress which should be taken into consideration in clinical work.

## Competing interests

The authors declare that they have no competing interests.

## Authors’ contributions

SK performed the statistical analysis and drafted the manuscript. MS participated in the design of the study and helped to draft the manuscript. SS conceived the study and helped to revise the manuscript. KM conceived the study, and participated in its design and coordination and helped to draft the manuscript. All authors have read and approved the final manuscript.

## Pre-publication history

The pre-publication history for this paper can be accessed here:

http://www.biomedcentral.com/1471-2296/13/107/prepub

## References

[B1] The Finnish Cancer Registry Institute for Statistical and Epidemiological Cancer Research Helsinki, Finland [ http://stats.cancerregistry.fi/stats/fin/vfin0008i0.html] (accessed December 2010)

[B2] Population Register Centre Helsinki, Finland http://www.vrk.fi/default.aspx?site=4] (accessed August 2012)

[B3] Korkeila K Suominen S Ahvenainen J Ojanlatva A Rautava P Helenius H Koskenvuo M Non-response and related factors in a nation-wide health survey Eur J Epidemiol 2001 17 11 991 999 10.1023/A:1020016922473 12380710

[B4] Suominen S Koskenvuo K Sillanmäki L Vahtera J Korkeila K Kivimäki M Mattila K Virtanen P Sumanen M Rautava P Koskenvuo M Non-response in a nationwide follow-up postal survey in Finland: a register-based mortality analysis of respondents and non-respondents of the Health and Social Support (HeSSup) Study BMJ Open 2012 2 e000657 10.1136/bmjopen-2011-000657 PMC3307122 22422917

[B5] Finnish Cancer Registry Institute for Statistical and Epidemiological Cancer Research: Cancer in Finland 2008 and 2009 Cancer Statistics of the National Institute for Health and Welfare (THL) 2011 Helsinki: Cancer Society of Finland Publication No. 84 [ www.cancer.fi/syoparekisteri/en/] (accessed August 2012)

[B6] The Finnish Cancer Registry Institute for Statistical and Epidemiological Cancer Research Helsinki, Finland [ http://www.cancer.fi/syoparekisteri/en/registration] (accessed August 2012)

[B7] Wilson PW Paffenbarger RS Jr Morris JN Havlik RJ Assessment methods for physical activity and physical fitness in population studies: report of a NHLBI workshop Am Heart J 1986 111 6 1177 1192 10.1016/0002-8703(86)90022-0 3716991

[B8] Reeder LG Schrama PG Dirken JM Stress and cardiovascular health: an international cooperative study I. Soc Sci Med 1973 7 8 573 584 4731468 10.1016/0037-7856(73)90026-7

[B9] Beck AT Ward CH Mendelson M Mock J Erbaugh J An inventory for measuring depression Arch Gen Psychiatry 1961 4 6 561 571 10.1001/archpsyc.1961.01710120031004 13688369

[B10] Koskenvuo M Kaprio J Rose RJ Kesäniemi A Sarna S Heikkilä K Langinvainio H Hostility as a risk factor for mortality and ischemic heart disease in men Psychosom Med 1988 50 4 330 340 3413267 10.1097/00006842-198807000-00002

[B11] Scheier MF Carver CS Bridges MW Distinguishing optimism from neuroticism (and trait anxiety, self-mastery, and self-esteem): a reevaluation of the Life Orientation Test J Pers Soc Psychol 1994 67 6 1063 1078 7815302 10.1037//0022-3514.67.6.1063

[B12] Koivumaa-Honkanen H Honkanen R Viinamäki H Heikkilä K Kaprio J Koskenvuo M Self-reported life satisfaction and 20-year mortality in healthy Finnish adults Am J Epidemiol 2000 152 10 983 991 10.1093/aje/152.10.983 11092440

[B13] Rahkonen O Lahelma E Huuhka M Past or present? Childhood living conditions and current socioeconomic status as determinants of adult health Soc Sci Med 1997 44 3 327 336 10.1016/S0277-9536(96)00102-5 9004368

[B14] Kivimäki M Vahtera J Elovainio M Lillrank B Kevin MV Death or illness of a family member, violence, interpersonal conflict, and financial difficulties as predictors of sickness absence: longitudinal cohort study on psychological and behavioral links Psychosom Med 2002 64 5 817 825 10.1097/01.PSY.0000031576.42041.B1 12271113

[B15] Romanow K Varjonen J Kaprio J Koskenvuo M Life events and depressiveness − the effect of adjustment for psychosocial factors, somatic health and genetic liability Acta Psychiatr Scand 2003 107 1 25 33 10.1034/j.1600-0447.2003.01419.x 12558538

[B16] Sarason IG Sarason BR Shearin EN Pierce GR A brief measure of social support: practical and theoretical implications J Soc Pers Relatsh 1987 4 4 497 510 10.1177/0265407587044007

[B17] Sumanen M Koskenvuo M Sillanmäki L Mattila K Childhood adversities experienced by working-aged coronary heart disease patients J Psychosom Res 2005 59 5 331 335 10.1016/j.jpsychores.2005.04.005 16253624

[B18] Korpimäki S Sumanen M Sillanmäki L Mattila K Cancer in working-age is not associated with childhood adversities Acta Oncol 2010 49 4 436 440 10.3109/02841860903521103 20121670

[B19] Teppo L Pukkala E Lehtonen M Data quality and quality control of a population-based cancer registry. Experience in Finland Acta Oncol 1994 33 4 365 369 10.3109/02841869409098430 8018367

[B20] Haapanen N Miilunpalo S Pasanen M Oja P Vuori I Agreement between questionnaire data and medical records of chronic diseases in middle-aged and elderly Finnish men and women Am J Epidemiol 1997 145 8 762 769 10.1093/aje/145.8.762 9126003

[B21] Hunziker S Schläpfer M Langewitz W Kaupfann G Nüesch R Battegay E Zimmerli U Open and hidden agendas of “asymptomatic” patients who request check-up exams BMC Fam Pract 2011 12 22 10.1186/1471-2296-12-22 21504617 PMC3094231

[B22] Grassi L Rosti G Psychosocial morbidity and adjustment to illness among long-term cancer survivors Psychosomatics 1996 37 6 523 532 10.1016/S0033-3182(96)71516-5 8942203

[B23] Parker PA Baile WF De Moor C Cohen L Psychosocial and demographic predictors of quality of life in a large sample of cancer patients Psychooncology 2003 12 2 183 193 10.1002/pon.635 12619150

[B24] Oerlemans ME van den Akker M Schuurman AG Kellen E Buntinx F A meta-analysis on depression and subsequent cancer risk Clin Prac Epidemiol Mental Health 2007 3 29 10.1186/1745-0179-3-29 PMC2235847 18053168

[B25] Strömberg R Backlund LG Löfvander M Psychosocial stressors and depression at a Swedisch primary health care centre A gender perspective study BMC Fam Pract 2011 12 120 10.1186/1471-2296-12-120 22047446 PMC3256103

[B26] Hamer M Yoichi C Molly GJ Psychological distress and cancer mortality J Psychosom Res 2009 66 3 255 258 10.1016/j.jpsychores.2008.11.002 19232239

[B27] Chida Y Hamer M Wardle J Steptoe A Do stress-related psychosocial factors contribute to cancer incidence and survival? Nature Clin Pract 2008 5 8 466 475 10.1038/ncponc1134 18493231

[B28] Pukkala E Weiderpass E Time trends in socio-economic differences in incidence rates of cancers of the breast and female genital organs (Finland, 1971–1995) Int J Cancer 1999 81 1 56 61 10.1002/(SICI)1097-0215(19990331)81:1<56::AID-IJC11>3.0.CO;2-4 10077153

[B29] Gustavsson-Lilius M Julkunen J Hietanen P Quality of life in cancer patients: The role of optimism, hopelessness, and partner support Qual Life Res 2007 16 1 75 87 10.1007/s11136-006-9101-4 17109191

[B30] Whitlock G Lewington S Sherliker P Clarke R Emberson J Halsey J Qizilbash N Collins R Peto R Prospective studies collaboration Body-mass index and cause-specific mortality in 900 000 adults: collaborative analyses of 57 prospective studies Lancet 2009 373 9669 1083 1096 10.1016/S0140-6736(09)60318-4 19299006 PMC2662372

[B31] Linsky A Nyambose J Battaglia TA Lifestyle behaviors in Massachusetts adult cancer survivors J Cancer Surviv 2011 58 1 27 34 21132395 10.1007/s11764-010-0162-6

[B32] Traeger L Penedo FJ Gonzalez JS Dahn JR Lechner SC Schneiderman N Antoni MH Illness perceptions and emotional well-being in men treated for localized prostate cancer J Psychosom Res 2009 67 5 389 397 10.1016/j.jpsychores.2009.03.013 19837201

[B33] Blomberg BB Alvarez JP Diaz A Romero MG Lechner CS Carver CS Holley H Antoni MH Psychosocial adaptation and cellular immunity in breast cancer patients in the weeks after surgery: An exploratory study J Psychosom Res 2009 67 5 369 376 10.1016/j.jpsychores.2009.05.016 19837199 PMC2764537

[B34] Baider L Ever-Hadani P Goldzweig G Wygoda MR Peretz T Is perceived family support a relevant variable in psychological distress? A sample of prostate and breast cancer couples J Psychosom Res 2003 55 5 453 460 10.1016/S0022-3999(03)00502-6 14581100

[B35] Shapiro SL Lopez AM Schwartz GE Bootzin R Figueredo AJ Braden CJ Kurker SF Quality of life and breast cancer: Relationship to psychosocial variables J Clin Psychol 2001 57 4 501 519 10.1002/jclp.1026 11255204

[B36] Lebel S Rosberger Z Edgar L Devins GM Predicting stress-related problems in long-term breast cancer survivors J Psychosom Res 2008 65 6 513 523 10.1016/j.jpsychores.2008.07.018 19027439

[B37] Haftgoli N Favrat B Verdon F Vaucher P Bischoff T Burnand B Herzig L Patients presenting with somatic complaints in general practice: depression, anxiety and somatoform disorders are frequent and associated with psychosocial stressors BMC Fam Pract 2010 11 67 10.1186/1471-2296-11-67 20843358 PMC2945969

